# A Global Multiregional Proteomic Map of the Human Cerebral Cortex

**DOI:** 10.1016/j.gpb.2021.08.008

**Published:** 2021-11-08

**Authors:** Zhengguang Guo, Chen Shao, Yang Zhang, Wenying Qiu, Wenting Li, Weimin Zhu, Qian Yang, Yin Huang, Lili Pan, Yuepan Dong, Haidan Sun, Xiaoping Xiao, Wei Sun, Chao Ma, Liwei Zhang

**Affiliations:** 1 Core Facility of Instrument, Institute of Basic Medical Sciences, Chinese Academy of Medical Sciences, School of Basic Medicine, Peking Union Medical College, Beijing 100005, China; 2 State Key Laboratory of Proteomics, Beijing Proteome Research Center, National Center for Protein Sciences (Beijing), Beijing Institute of Lifeomics, Beijing 102206, China; 3 Department of Neurosurgery, China National Clinical Research Center for Neurological Diseases, Beijing Tiantan Hospital, Capital Medical University, Beijing 100050, China; 4 Department of Human Anatomy, Histology, and Embryology, Institute of Basic Medical Sciences, Neuroscience Center, Chinese Academy of Medical Sciences, School of Basic Medicine, Peking Union Medical College, Beijing 100005, China

**Keywords:** Brodmann area, Proteomics, Cerebral cortex, Cingulate cortex, Sensorimotor cortex

## Abstract

The Brodmann area (BA)-based map is one of the most widely used cortical maps for studies of human brain functions and in clinical practice; however, the molecular architecture of BAs remains unknown. The present study provided a global multiregional proteomic map of the human **cerebral cortex** by analyzing 29 BAs. These 29 BAs were grouped into 6 clusters based on similarities in proteomic patterns: the motor and sensory cluster, vision cluster, auditory and Broca’s area cluster, Wernicke’s area cluster, **cingulate cortex** cluster, and heterogeneous function cluster. We identified 474 cluster-specific and 134 BA-specific signature proteins whose functions are closely associated with specialized functions and disease vulnerability of the corresponding cluster or BA. The findings of the present study could provide explanations for the functional connections between the anterior cingulate cortex and **sensorimotor cortex** and for anxiety-related function in the sensorimotor cortex. The brain transcriptome and proteome comparison indicates that they both could reflect the function of cerebral cortex, but show different characteristics. These proteomic data are publicly available at the Human Brain Proteome Atlas (www.brain-omics.com). Our results may enhance our understanding of the molecular basis of brain functions and provide an important resource to support human brain research.

## Introduction

The human cerebral cortex, the outer layer of the cerebrum, exhibits great complexity in its histological structure and cellular organization [1]. In 1909, Korbininan Brodmann distinguished a total of 52 subareas based on the differences in cytoarchitectural organization across the cortex; these areas were later termed Brodmann areas (BAs) [2]. Subsequently, BA-based map has become one of the most widely used cortical maps for the investigation of brain functions and in clinical practice. BAs are often correlated with certain cortical functions. For example, Broca’s speech and language area is consistently localized in BAs 44 and 45. Cytoarchitecture [1], [2], imaging [1], [3], and functional roles [3] of BAs have been extensively studied, and comprehensive human brain Magnetic Resonance Imaging (MRI) [1], histology [1], and connectome maps [3] have been investigated.

Molecular annotations, especially at the protein level, of various BAs will provide a deeper understanding of the molecular basis for diverse brain functions. Transcriptomic and proteomic maps of the brain have been generated to study the molecular basis of distinct cytoarchitecture and functions of various BAs [1]. The Allen Institute for Brain Science has generated a transcriptional atlas of the human brain by comprehensive profiling of nearly 900 anatomically precise subdivisions using microarrays [1]. The human neocortex is characterized by a relatively homogeneous transcriptional pattern; the molecular topography of the neocortex is similar to its cytoarchitecture. At the protein level, several studies generated a protein atlas of the mouse brain and identified some brain region-restricted proteins [4], [5] or cell type-specific proteins [4]. In 2014, Kim et al. drafted a proteomic profile of the human frontal cortex from a pool of three individuals and identified 9060 proteins [6]. In 2017, Becky et al. [7] performed a proteomic survey of 7 postnatal human brain regions, including two cerebral cortex regions, the cerebellar cortex, the hippocampus, and other neurological nuclei, ranging from early infancy to adulthood. Individual variations due to age and sex were considerably lower than variations between brain regions. Importantly, the differences in brain cytoarchitecture, development, and function are better represented by changes at the protein level than by changes at the transcriptional level.

Previous omics studies have increased our understanding of brain function. However, some important scientific aspects have not been investigated. Functional annotation of the brain demonstrated that several brain regions are functionally connected but are not spatially adjacent, such as BA41/42 (auditory cortex) and BA44/45 (Broca’s area and language production) [3], [8]. However, the data on the brain transcriptome showed a relatively homogeneous expression pattern in the neocortex, and molecular topography in this area is similar to spatial topography. Proteins are the main functional operators in all cells. The proteomic map of cerebral cortex may provide new insight into this scientific context. Previous studies of the brain proteome investigated only a few BAs, which are not sufficient to illustrate these aspects. Thus, a comprehensive proteomic map of various BAs of the human cerebral cortex is urgently needed.

The present study used 29 BA postmortem specimens to generate a global multiregional proteomic map of the human cerebral cortex. Initially, an extensive proteomic library of BA46 was generated. Then, 29 BAs were quantitatively analyzed using the two-dimensional liquid chromatography coupled with tandem mass spectrometry (2DLC-MS/MS) approach combined with isobaric tags for relative and absolute quantitation (iTRAQ) labeling. Clustering analysis of 29 BAs was performed based on the similarities in protein expression patterns. Cluster- and BA-specific signature proteins were identified and analyzed using bioinformatics. The major functional brain areas, including the cingulate cortex, motor cortex, sensory cortex, language area, and visual cortex, were functionally annotated using proteomic data. The transcriptomes of the aforementioned BAs were also analyzed, and the comparison of proteome and transcriptome on BA functions was presented. The results of the present study provide a molecular basis for BA functions. Furthermore, the database provides an important resource to support brain research.

## Results

### A broad and comprehensive atlas of the brain proteome

**Supplementary figure 1:**
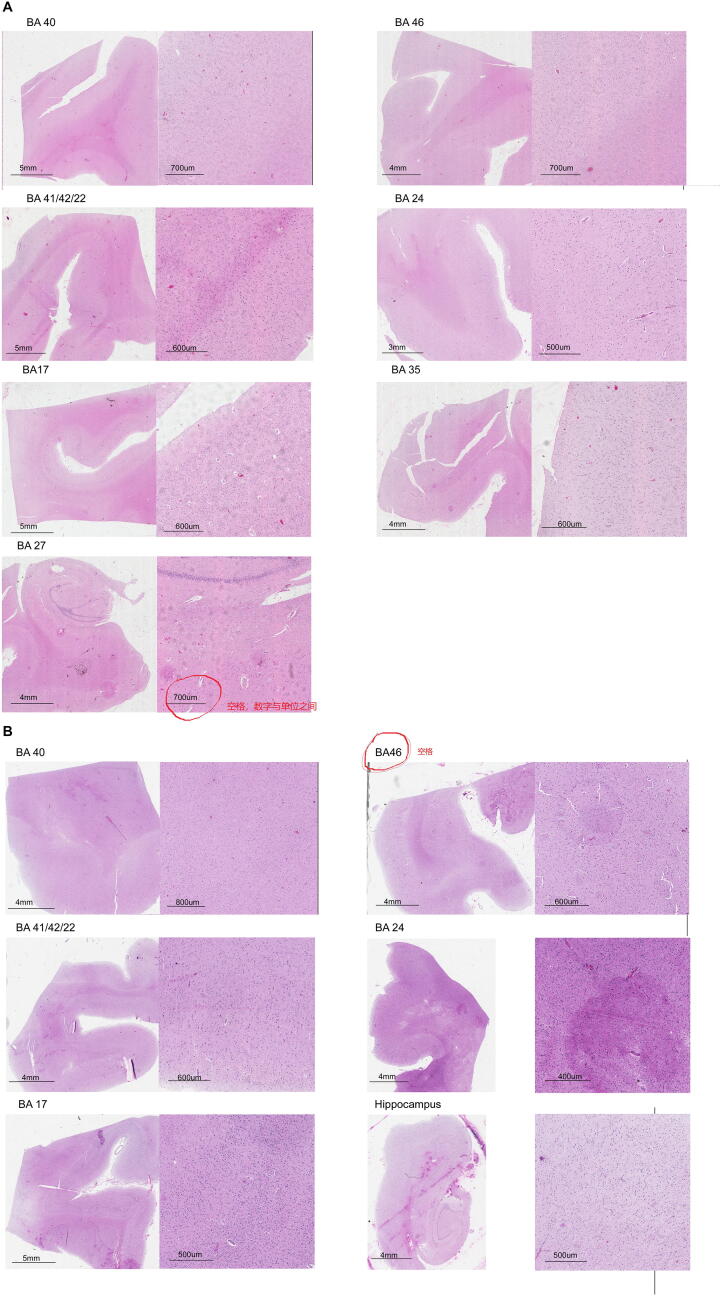
The HE staining data of two brains. **A**. Brain 1. **B**. Brain 2. BA, Brodmann area.

A single human brain specimen was obtained from the National Human Brain Bank for Development and Function, Chinese Academy of Medical Sciences and Peking Union Medical College, Beijing, China. The brain specimen was collected from a 54-year-old willed male donor who died of lung cancer and did not have any history of brain metastasis, trauma, or other diseases. The samples of 29 BAs of the left cerebral hemisphere were collected with a postmortem delay of less than 6 h. Hematoxylin-eosin (HE) staining of the samples showed that the brain was cancer-free (Figure S1). The protocols for brain tissue acquisition, dissection, and sample preparation were under consistent and stringent quality control (see Materials and methods) based on the standardized operational protocol (SOP) of the Human Brain Bank of China [9].

**Figure 1:**
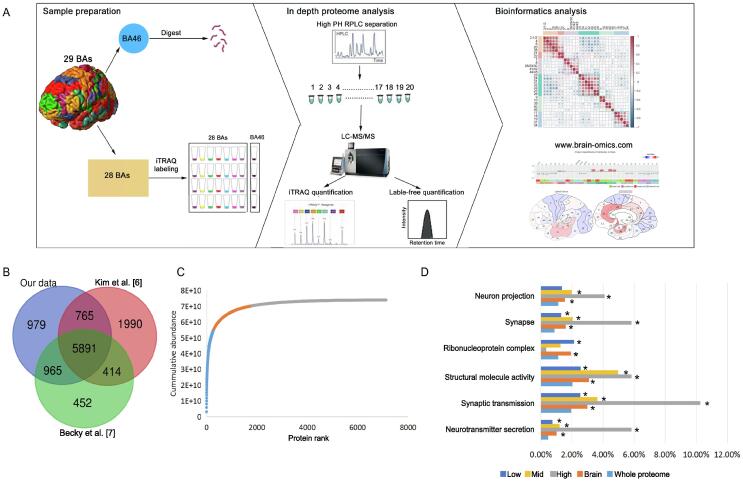
**A broad and comprehensive atlas of brain proteome covering 29 BAs** **A.** Graphical illustration of the workflow for generating a brain proteome database in BA46 and a brain proteomic map in the 29 major BAs. First, an extensive proteomic library was generated for BA46 by 2DLC-MS/MS and label-free quantification. Then, 29 BAs were quantitatively analyzed by 2DLC-MS/MS and iTRAQ quantification. Further, a website, Human Brain Proteome Atlas (HBPA; www.brain-omics.com), was built. **B.** Venn diagram showing the overlap of proteins of BA46 identified in the present study and previous studies [6], [7]. **C.** Accumulation of protein mass from the highest-abundance to the lowest-abundance protein in BA46. The high-abundance proteins (gray, top for 75% of total protein abundance), mid-abundance proteins (orange, top 75%–95% of total protein abundance), and low-abundance proteins (blue, bottom 5% total protein abundance) were shown by distinct colors. **D.** GO enrichment analysis of the brain proteome as well as the high-, mid-, and low-abundance brain proteins compared with the whole proteome. The percentages of the proteins in each category were shown in the bar plot. *, *P* < 0.05. BA, Brodmann area; 2DLC-MS/MS, two-dimensional liquid chromatography coupled with tandem mass spectrometry; RPLC, reverse phase liquid chromatography; iTRAQ, isobaric tags for relative and absolute quantitation; GO, Gene Ontology.

**Supplementary figure 2:**
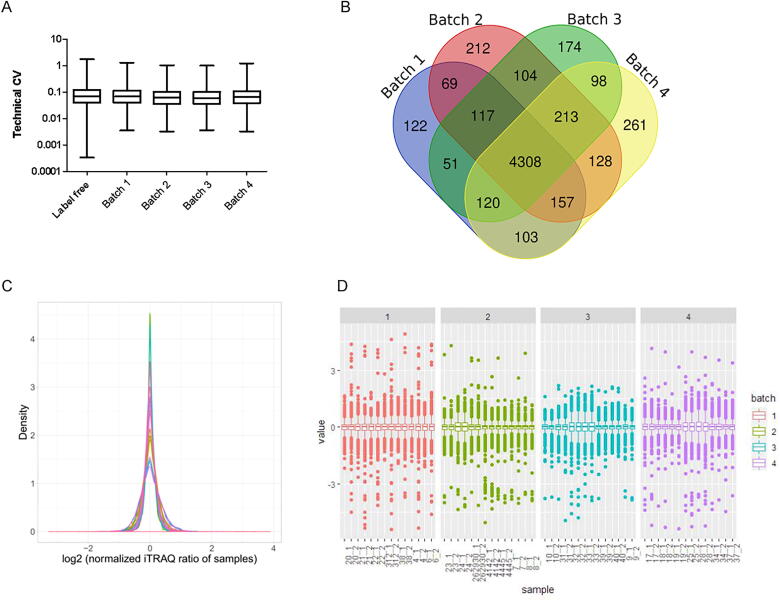
Reliability of iTRAQ analysis pipeline. **A**. Technical CV distributions of label-free and iTRAQ quantification. **B**. The Venn diagram of the proteins quantified in 4 batches. **C**. Log ratio distribution of iTRAQ quantification results. **D**. The ratio distributions of each run in 4 batches. iTRAQ, isobaric tags for relative and absolute quantitation.

BA46 [dorsolateral prefrontal cortex (DFC)] plays an important role in many cognitive processes and had been comprehensively characterized in previous proteomic studies [6], [7]. Therefore, deep proteomic profiling of BA46 was performed using the 2DLC-MS/MS approach (Figure 1A) to construct a Chinese reference brain protein library. A total of 8600 proteins (Table S1; data quality in Figure S2A) were identified in the present study, corresponding to 73% and 89% of the proteins identified in the studies of Kim et al. [6] and Becky et al. [7], respectively. Additional 979 (11.4%) proteins were identified by brain proteome analysis for the first time (Figure 1B; Table S1). Thus, we generated a broad and high-coverage proteomic dataset with identification depth similar to that achieved by the two most comprehensive datasets reported in previous studies [6], [7].

**Supplementary figure 3:**
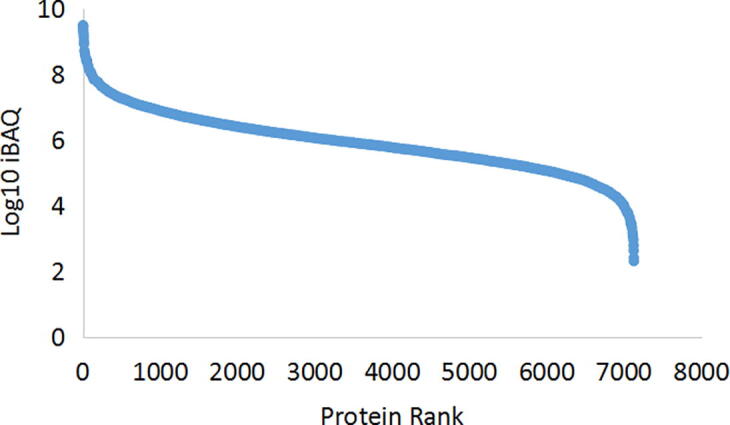
Protein abundance distribution of BA46 from the highest abundance to the lowest abundance protein.

Then, to provide a comprehensive protein abundance analysis, the proteome abundance in BA46 was calculated by the intensity-based absolute quantification (iBAQ) method [10]. A total of 7129 proteins were successfully quantified, and their abundances spanned 7 orders of magnitude (Figure S3). Approximately 4% of the most abundant proteins (292 proteins) accounted for 75% of the total protein abundance and were considered as high-abundance brain proteins in the present study. The remaining 20% and 5% of the total protein abundance corresponded to 1513 mid-abundance proteins and 5324 low-abundance proteins, respectively (Figure 1C; Table S1). Gene Ontology (GO) analysis revealed that nervous system structure and function were enriched in this brain proteome, especially in the case of high-abundance proteins (Figure 1D; Table S1), implying the important role of high-abundance proteins in brain functions.

Finally, to investigate specific proteomic profiling in BAs and explore the associations between BA protein expression and function, we used iTRAQ-based quantitative proteomics to generate a region-resolved proteome map of the brain covering 29 BAs with important significance for research and clinical applications (Figure 1A; Table 1). A total of 4308 proteins were quantified in all BAs (Table S2). The batch effect of four batches was evaluated by qualitative and quantitative analyses. The median Coefficient of Variation (CV) of four batches was around 6% (Figure S2A), and the Venn diagram of protein identification in four batches (Figure S2B) showed high overlapping. The log-ratio distributions of 29 iTRAQ quantification results (Figure S2C) showed similar normal distribution, and the detailed ratio distributions of each BA in four batches (Figure S2D) showed similar distributions.

**Table 1:** **Location, function,and proteomic clustering of 29 human brain BAs**

	**BA**	**Lobe**	**Location**	**Function**	**Refs.**
Motor and sensory	3/1/2	Parietal lobe	Postcentral gyrus	Somatosensory system	[72]
	4	Frontal lobe	Precentral gyrus	Primary motor cortex	[73]
	6	Frontal lobe	Frontal lobe	Premotor cortex and supplementary motor cortex	[73]
	20	Temporal lobe	Inferior temporal gyrus	Visual processing and recognition	[74]
	38	Temporal lobe	Temporal pole	Visceral emotional responses	[75]
Vision	17	Occipital lobe	Occipital lobe	Visual processing	[76]
	18	Occipital lobe	Occipital lobe	Visual processing	[76]
	19	Occipital lobe	Occipital lobe	Visual processing	[76]
	37	Temporal lobe	Fusiform gyrus	Visual recognition	[13], [14], [15]
	34	Temporal lobe	Parahippocampal gyrus	Neuron information processing, visual processing	[16]
Auditory and Broca’s area	8	Frontal lobe	Prefrontal cortex	Eye movements, writing language	[17]
	7	Parietal lobe	Parietal lobe	Visuo-motor coordination, speech and language	[18]
	26/29/30	Cingulate cortex	Retrosplenial cortex	Cognition, memory, auditory	[19], [77]
	41/42	Temporal lobe	Anterior transverse temporal gyrus	Auditory cortex	[78]
	44/45	Frontal lobe	Inferior frontal gyrus	Broca’s area, language production	[79]
Heterogeneous function	9	Frontal lobe	Dorsolateral prefrontal cortex	Executive functions, cognition, behavior	[80]
	10	Frontal lobe	Anterior prefrontal cortex	Executive functions, cognition, memory	[81]
	39	Parietal lobe	Angular gyrus	Complex language function, mathematics processing, and spatial cognition	[82]
	40	Parietal lobe	Supramarginal gyrus	Language perception and processing, visual word recognition	[83]
	46	Frontal lobe	Dorsolateral prefrontal cortex	Executive functions, cognition, behavior	[80]
Wernicke’s area	21	Temporal lobe	Middle temporal gyrus	Wernicke’s area, comprehension of written and spoken language	[20]
	22	Temporal lobe	Superior temporal gyrus	Wernicke’s area, comprehension of written and spoken language	[20]
Cingulate cortex	31	Cingulate cortex	Posterior cingulate cortex	Emotion and memory, intrinsic control networks	[24]
	32	Cingulate cortex	Anterior cingulate cortex	Emotional and cognitive processing	[84], [85]
	33	Cingulate cortex	Anterior cingulate cortex	Emotional and cognitive processing	[84], [85]
	23	Cingulate cortex	Ventral posterior cingulate cortex	Emotion and memory, intrinsic control networks	[24]
	24	Cingulate cortex	Ventral anterior cingulate cortex	Emotional and cognitive processing	[84], [85]
	25	Cingulate cortex	Subgenual cingulate	Emotion and memory	[25]
	28	Temporal lobe	Ventral entorhinal cortex	Neuron information processing, memory	[26]

*Note*: BA, Brodmann area.

**Supplementary figure 4:**
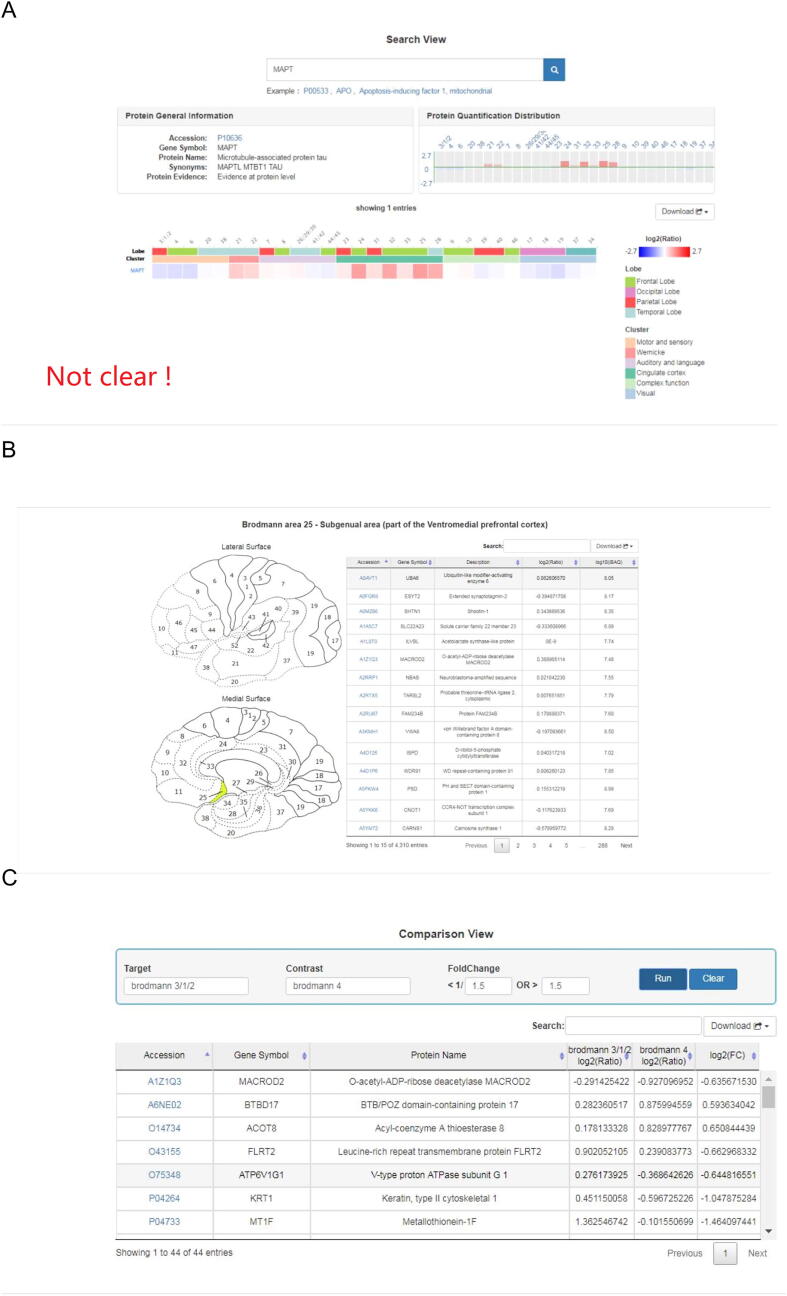
Data visualization in Human Brain Proteome Atlas website. **A**. Protein quantification distribution of MAPT in 29 Brodmann areas. **B**. Protein expression data in Brodmann area 25 (Subgenual area). **C**. Comparison of the differential proteins between BA3/1/2 and BA4.

A total of 1241 proteins were characterized by interarea variability (the difference between the maximum and minimum ratios ≥ 2, detailed in Materials and methods). The Human Brain Proteome Atlas (HBPA; www.brain-omics.com) was constructed to enhance visualization and use of these data, which are publicly accessible (Figure S4).

### Brain proteome atlas reflects functional parcellations

**Figure 2:**
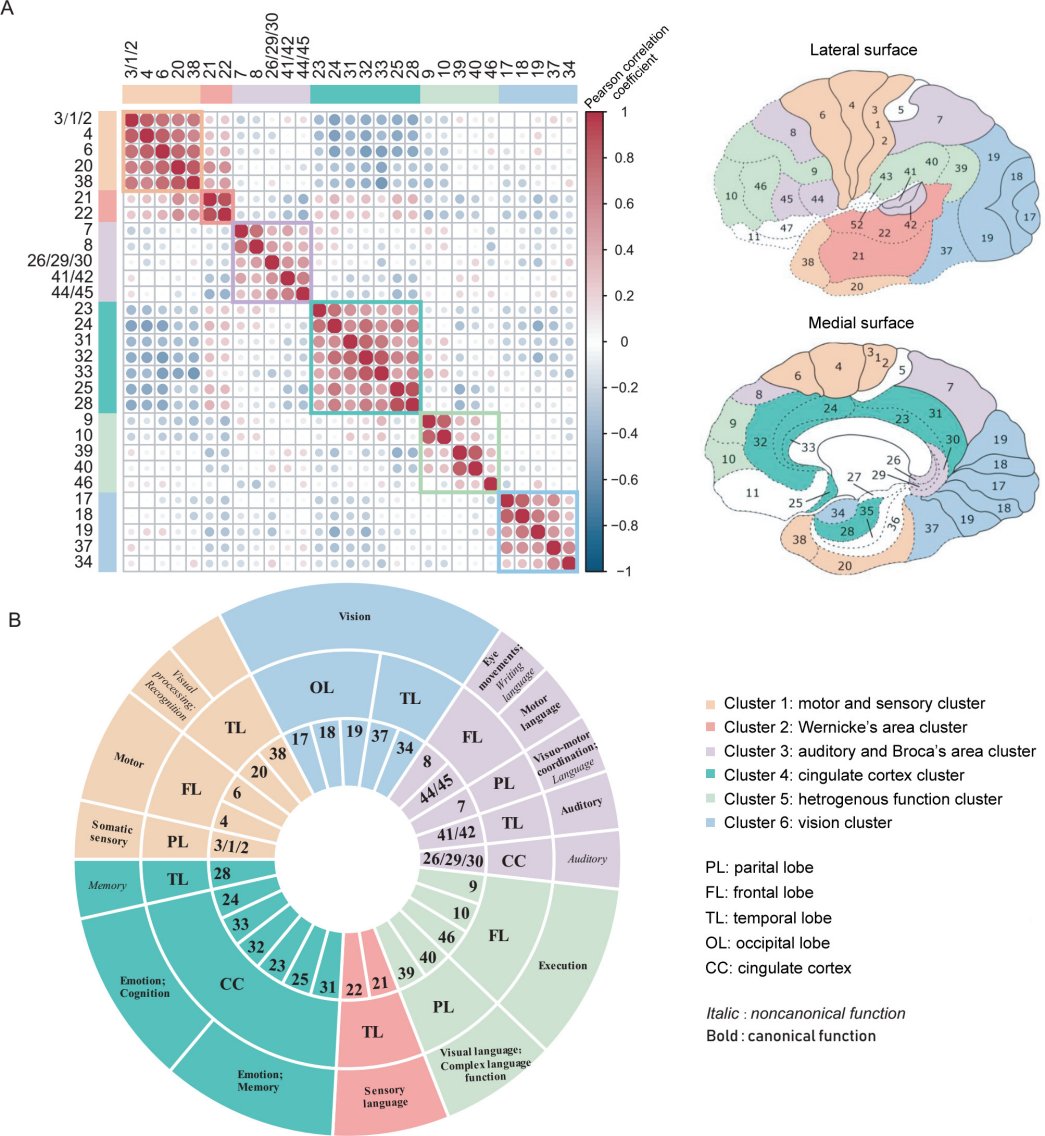
**Brain proteome atlas reflectsfunctional parcellations** **A.** The 29 brain areas were divided into 6 clusters, including motor and sensory cluster, vision cluster, auditory and Broca’s area cluster, Wernicke’s area cluster, cingulate cortex cluster, and heterogeneous function cluster. The Pearson correlation heatmap of 29 BAs was shown on the left. Red color indicates positive correlation; blue color indicates negative correlation. The locations of 29 BAs were shown on the right. **B.** Brief annotations of 29 BAs in the 6 clusters. The lobe location of each BA was annotated in the inner circle, and the function of each BA was annotated in the outer circle.

**Supplementary figure 5:**
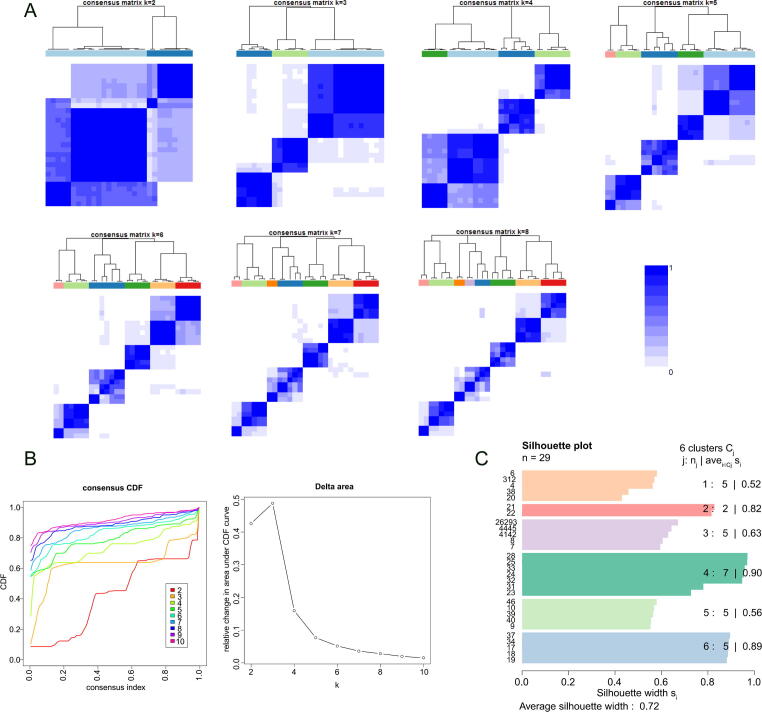
The protein clustering analysis. **A**. The clustering is derived by consensus clustering based on 1000 resampled data sets, exploring the range from k=2 to k=8. **B**. Based on both the consensus CDF area curve and the delta plot assessing change in CDF area, the CDF of consensus increased at greater k values. At k = 6, a relative increase in CDF reached a very small value. **C**.Silhouette plots were performed to evaluate the stability of theclustering. The silhouette plot at k = 6 showed a mean silhouette score of 0.72, and all scores were positive. CDF, cumulative distribution function.

Proteins are the main components and functional operators in the cells; thus, BAs with similar protein expression patterns may have similar cytoarchitecture or perform similar functions. Based on the similarities of protein expression patterns, *k*-means clustering [11] was used for the classification of BAs. The consensus cumulative distribution function (CDF) plot and silhouette plot (Figure S5) based on the consensus matrix were used to group 29 BAs into 6 clusters (detailed data in Materials and methods; Figure 2A; Table 1).

The cingulate cortex cluster (cluster 4) included BAs in the cingulate cortex, subgenual cingulate cortex, and entorhinal cortex. All these BAs belong to the transition areas between the allocortex (3 layers of neuronal cell bodies) and neocortex (6 layers of neuronal cell bodies). Therefore, the cytoarchitecture of the cingulate cortex cluster was different from that of the neocortex [12]. At the proteome level, the cingulate cortex cluster was characterized by protein patterns distinct from those of other clusters, which were mainly located in the neocortex (Figure 2A), implying that the brain proteome can reflect the differences in cytoarchitecture between the cingulate cortex and the neocortex.

The remaining 23 BAs within the neocortex were grouped into 5 clusters, and BAs within a single cluster were functionally similar. For example, the vision cluster (cluster 6) comprised a group of BAs (BA17, BA18, BA19, BA37, and BA34) engaged in visual processing (Figure 2B; Table 1). BA17, BA18, and BA19 are located in the occipital lobe, and BA37 is adjacent to BA19. All four BAs correspond to classic visual processing regions [13], [14], [15]. BA34 is located in the parahippocampal gyrus and is not spatially connected to other four BAs, although BA34 was reported to be related to visual processing. Another example is the auditory and Broca’s area cluster, which is composed of several spatially separated but functionally related BAs (Figure 2B). BA44/45 (Broca’s area and motor language area), BA8 (writing language area) [17], and BA7 (engaged in speech [18]) are directly involved in language processing, whereas BA41/42 (auditory cortex) and BA26/29/30 (engaged in sound processing) [19] are associated with auditory processing, which is indirectly linked to language processing [3], [8].

**Supplementary figure 6:**
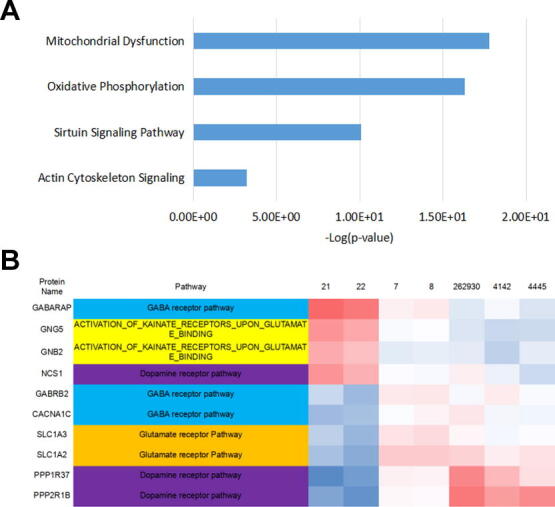
Analysis of the differential proteins between Wernicke’s area cluster and auditory and Broca’s area cluster. **A**. IPA-based canonical pathway analysis of differential proteins. The −log (Pvalue) of the canonical pathway terms were shown. **B**. Heatmap of differential proteins involved in language eloquent regions related to neurotransmitter pathways. The normalized protein expression value z-score represented the protein abundance. Red,high abundance. Blue, low abundance.

Interestingly, two language processing BAs, BA22 (classic Wernicke’s area and sensory language area) and adjacent BA21 (also considered as a part of Wernicke’s area [20]), were clustered into the Wernicke’s area cluster. This cluster was characterized by proteomic patterns distinct from the auditory and Broca’s area cluster. To illustrate the differences in protein expression between these two clusters, we compared differential proteins between the two clusters. A total of 88 and 108 proteins were expressed at higher levels in the Wernicke’s area cluster and auditory and Broca’s area cluster, with a fold change (FC) of 1.5, respectively (Table S3). Differentially expressed proteins were enriched in metabolic pathways, suggesting metabolic differences between the two language regions (Figure S6A). These proteins are also related to neurotransmitter pathways [21], including glutamate, γ-aminobutyric acid (GABA), and dopamine pathways (Figure S6B) [21]. In particular, the GABA receptor was expressed at a higher level in the auditory and Broca’s area cluster compared to that in the Wernicke’s area cluster in agreement with previous reports on an important role of the GABA receptor in the physiological functions of Broca’s area. An inhibitor of the GABA receptor causes dysfunction in Broca’s area and induces stuttering [22], and a GABA agonist improves chronic Broca’s aphasia [21]. Therefore, these differentially expressed proteins provide valuable information about the intrinsic mechanism of language processing.

### Cluster/BA-specific signature proteins are linked to the specialized functions of clusters/BAs

**Figure 3:**
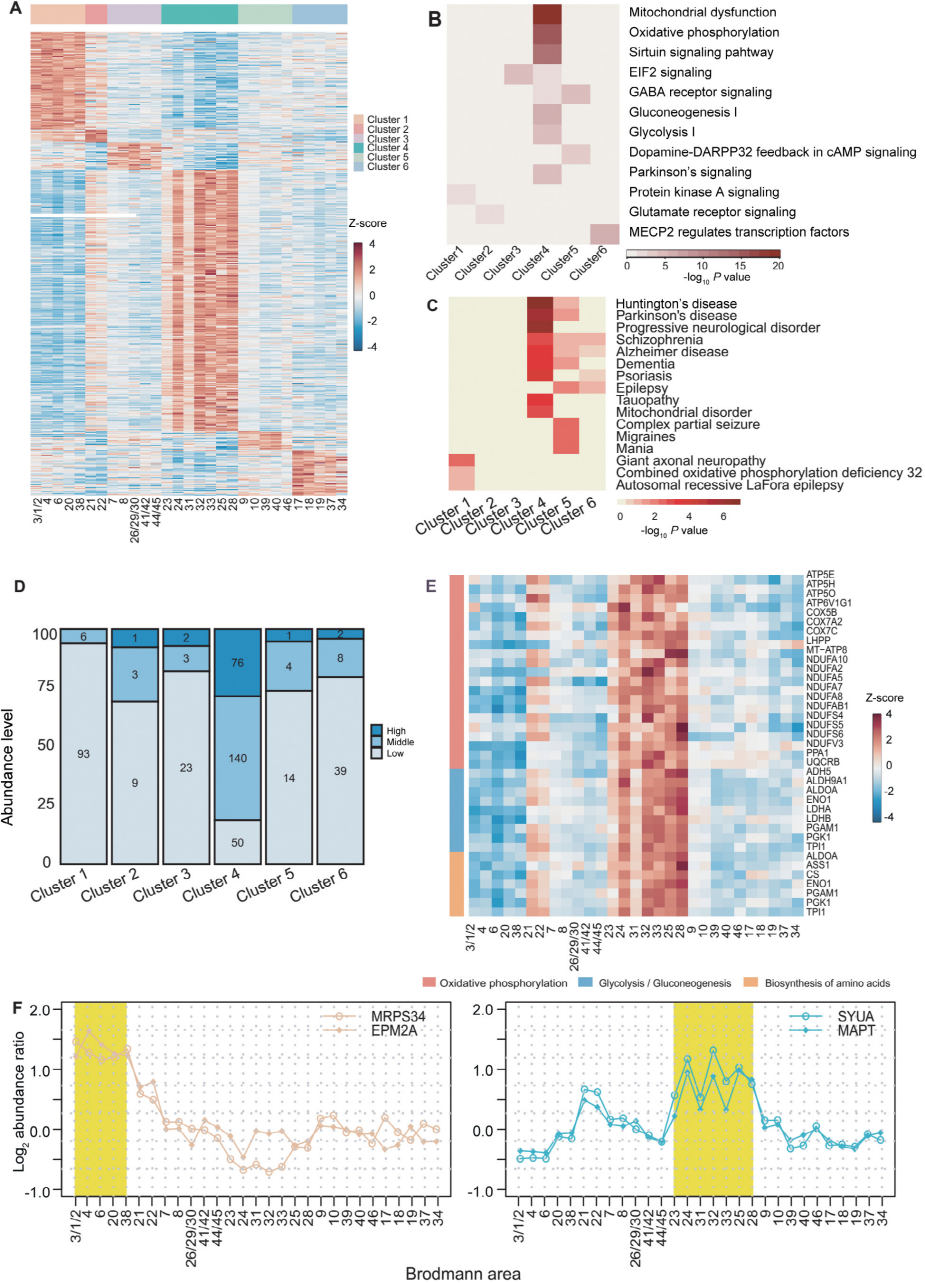
**Cluster-specific signature proteins are linked to the specialized function of the cluster** **A.** Heatmap of the cluster-specific signature proteins. The normalized protein expression value z-score represents the protein abundance. Red color indicates high abundance, and blue color indicates low abundance. Pathway (**B**) and disease (**C**) enrichment analyses of cluster-specific signature proteins. The −log_10_*P* value by two-sided hypergeometric test was shown in heatmaps. Red color indicates significantly enriched. **D.** Distribution of the high-, mid-, low-abundance proteins in the cluster-specific signature proteins. **E.** Heatmap of cingulate cortex cluster-specific signature proteins involved in glycolysis/gluconeogenesis, oxidative phosphorylation, and biosynthesis of amino acids. The normalized protein expression value z-score represents the protein abundance. Red color indicates high abundance, and blue color indicates low abundance. **F.** The relative expression levels of two motor and sensory cluster-specific signature proteins (MRPS34 and EMP2A) and two cingulate cortex cluster-specific signature proteins (SYUA and MAPT) in all the 29 BAs. The motor and sensory cluster and the cingulate cortex cluster were highlighted in yellow in the left and right panels, respectively.

**Supplementary figure 7:**
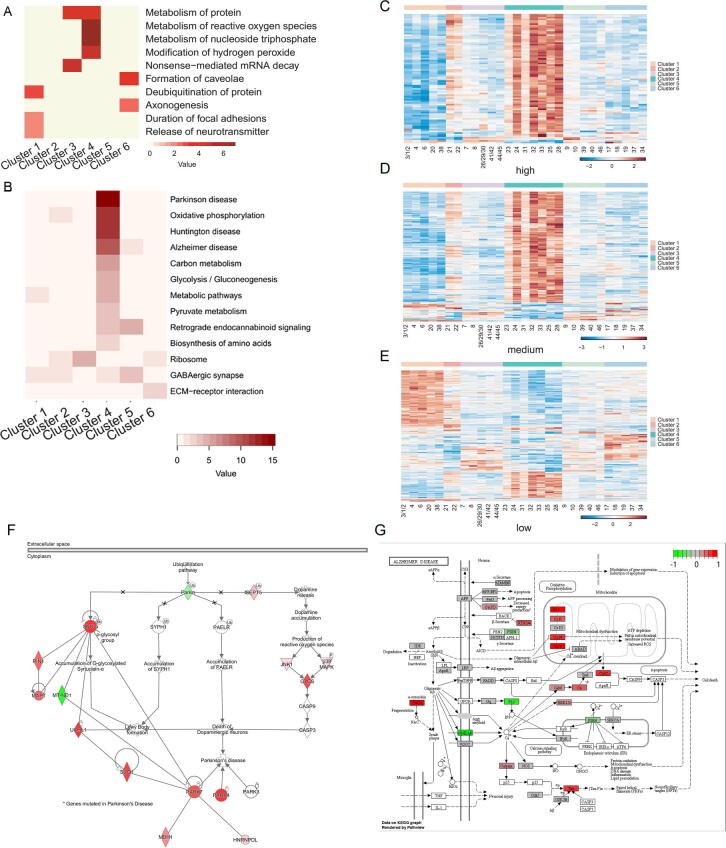
Functional analysis of cluster signature proteins, related to Figure 3. The IPA-based biofunction (**A**) and KEGG database-based pathway (**B**) enrichment analysis of cluster signature proteins. The −log (Pvalue) by two-sided hypergeometric test were shown in heatmap. Red, significantly enriched. Heatmap of the high-abundance proteins (**C**), mid-abundance proteins (**D**), and low abundance proteins (**E**) in the 29 BAs. The normalized protein expression value z-score represented the protein abundance. Red, high abundance. Blue, low abundance. **F**. Protein expression analysis of cingulate cortex cluster in Parkinson’s signaling. **G**. Protein expression analysis of cingulate cortex cluster in Alzheimer’s disease signaling. (Red, highly-expressed in cingulate cortex cluster; Green, lowly-expressed in cingulate cortex cluster).

BAs with similar protein expression patterns tend to have similar cytoarchitecture and functions; thus, cluster-specific signature proteins (proteins that were expressed at a high level in a certain cluster) may provide information about the specialized functions of a cluster. Iterative signature analysis (ISA; details described in Materials and methods) [23] was used to identify a total of 474 cluster-specific signature proteins in the present study (Figure 3A; Table S4). Pathway, function, and disease annotations of these proteins are shown in Figure 3B and C, Figure S7A and B, and Table S4.

**Supplementary figure 8:**
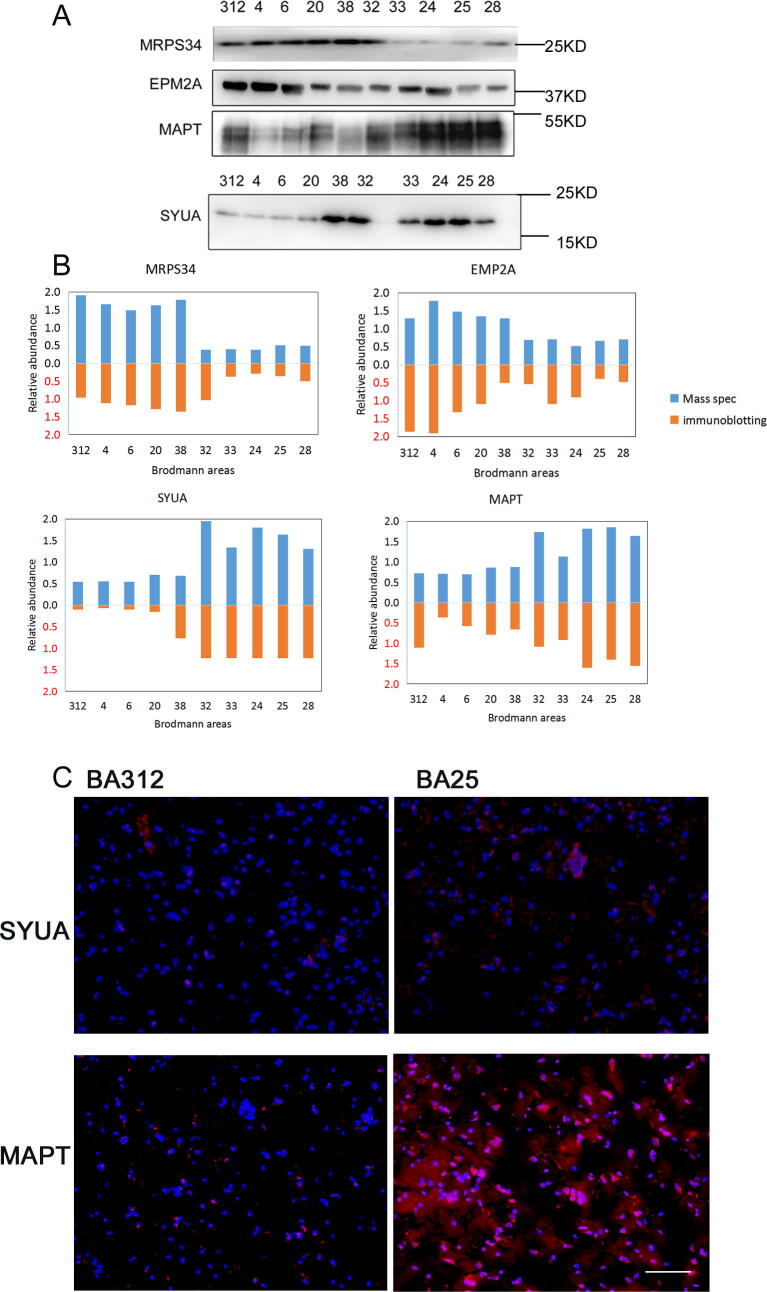
The Western Blot and IHC-Fr results of cluster signature proteins. **A**. Western Blot results of two motor and sensory cluster signature proteins (MRPS34 and EPM2A) and two cingulate cortex cluster signature proteins (MAPT and SYUA). **B**. Comparison between immunoblotting results and iTRAQ results of the above proteins. **C**. IHC-Fr validation results of two cingulate cortex cluster signature proteins (MAPT and SYUA). Scale bars represent 50 µm.

The cingulate cortex cluster had the largest number of signature proteins, 266 out of all 474 signature proteins. These proteins included 76 (26.3%) and 140 (52.6%) high- and mid-abundance proteins, respectively (Figure 3D, Figure S7C–E), corroborating the results regarding the differences in cytoarchitecture and protein expression between the cingulate cortex cluster and the neocortex. Functional analysis showed that these signature proteins are enriched in metabolic pathways, including important enzymes for oxidative phosphorylation (NADH dehydrogenase), glycolysis (ALDOA, PGK1, PGAM1, and ENOA), glycogen turnover (TPIS), and amino acid synthesis (ASS1 and PDHX) (Figure 3E), indicating high metabolic activity of the cingulate cortex. These results are in agreement with the metabolic analysis of the cingulate cortex reported in a previous study [24]. A total of 21 signature proteins were involved in cognition, emotion, and memory (Table S4), consistent with the functional characteristics of the cingulate cortex [24], [25], [26]. These results are in agreement with the functional characteristics of the cingulate cortex. Disease annotations indicated that proteins related to neurological degenerative diseases (Huntington’s disease, Parkinson’s disease, and Alzheimer’s disease) were enriched in the signature proteins of the cingulate cortex cluster. Several key proteins associated with signaling in Alzheimer’s and Parkinson’s diseases were expressed at high levels in the cingulate cortex cluster (Figure S7F and G). These proteins include tau (MAPT) and alpha-synuclein (SYUA), which are important biomarkers for Alzheimer’s disease [27], [28] and Parkinson’s disease [29], respectively (Figure 3F). The pathological accumulation of these two proteins is etiologically correlated with the development of Alzheimer’s [28] and Parkinson’s diseases [29]. The data of Western blot analysis showed that the protein expression patterns of MAPT and SYUA were consistent with the patterns detected by proteomic analysis (Figure S8A and B). The results of immunohistochemistry staining of frozen sections (IHC-Fr) also demonstrated that MAPT and SYUA were expressed at a high level in BA25 compared to that in BA4 (Figure S8C). The results of this study demonstrated the regional distribution of MAPT and SYUA for the first time, adding new information about the pathogenesis and vulnerability of these two neurodegenerative diseases in the cingulate cortex. Furthermore, another brain (brain 2) was used to validate the cluster-specific signature proteins by parallel reaction monitoring (PRM). Detailed clinical information on brain 2 is provided in Materials and methods. PRM analysis validated that 28 signature proteins were expressed at a high level in the cingulate cortex cluster (Table S5).

The motor and sensory cluster had 99 signature proteins. These proteins were enriched in the protein kinase A (PKA) pathway (Figure 3B). The PKA pathway is associated with brain motor function (neuronal plasticity in motor neurons [30], [31]) and sensation (pain sensitization in sensory neurons [32], [33]). Striatal-enriched protein tyrosine phosphatase (STEP), a molecule downstream of PKA, is associated with motor skill learning in the motor cortex [34]. Other signature proteins with potentially interesting functions include the neuron survival protein EMP2A and mitochondrial 28S ribosomal protein S34 (MRPS34) (Figure 3F). The functional loss of EMP2A was reported to cause sensorimotor cortex excitability in Lafora disease [35]. MRPS34 mutation causes delayed psychomotor development and neurodevelopmental deterioration in Leigh syndrome [36]. The immunological analysis confirmed that EMP2A and MRPS34 were expressed at a high level in the motor and sensory cluster (Figure S8). Two signature proteins of motor and sensory cluster, EMP2A and PTN5, were validated by PRM (Table S5).

The vision cluster included 49 signature proteins enriched in the methyl-CpG-binding protein 2 (MECP2)-regulated transcription factor pathway. MECP2 and transcriptionally regulated transcription factors are involved in the neurological system and neuropsychiatric disorders [37] (Figure 3B). MECP2 plays an important role in the normal development of the central nervous system and the maintenance of neurological functions. Mutation of the *MECP2* gene leads to Rettsyndrome, which is an autism spectrum-associated disorder with visual impairment [38]. In a mouse model, knockout of *MECP2* causes a progressive dysfunction of the visual cortex and rapid regression of visual acuity [39]. Three signature proteins of the vision cluster, MECP2, Transgelin (TAGLN), and CAVN1, were confirmed to be expressed at a high level by PRM (Table S5). Additionally, Becky et al. [7] reported a proteomic comparison of BA17 (primary visual cortex) and BA46 (DFC) in 11 individuals ranging from early infancy to adulthood. By comparing the 49 vision cluster-specific signature proteins in the present study with Becky’s study, 16 were expressed at a high level in the primary visual cortex in both studies (Table S5).

**Figure 4:**
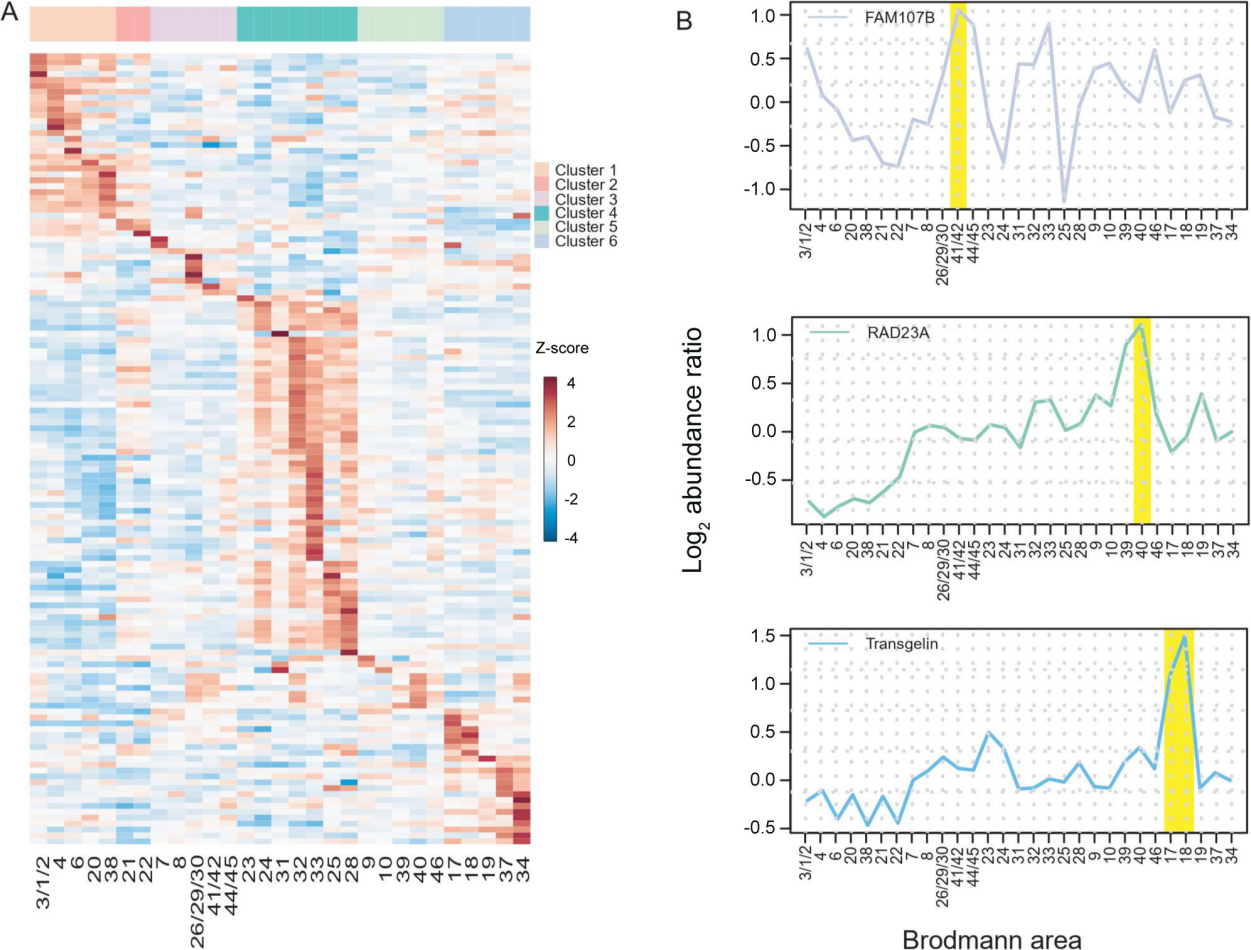
**Proteomic analysis of BA-specificsignature proteins** **A.** Heatmap of the BA-specific signature proteins in 29 BAs. The normalized protein expression value z-score represents the protein abundance. Red color indicates high abundance, and blue color indicates low abundance. **B.** The relative expression levels of three BA-specific signature proteins, TAGLN, RAD23A, and FAM107B, in 29 BAs. The BA41/42, BA40, BA17, and BA18 were highlighted in yellow in the upper, middle, and lower panels, respectively.

**Supplementary figure 9:**
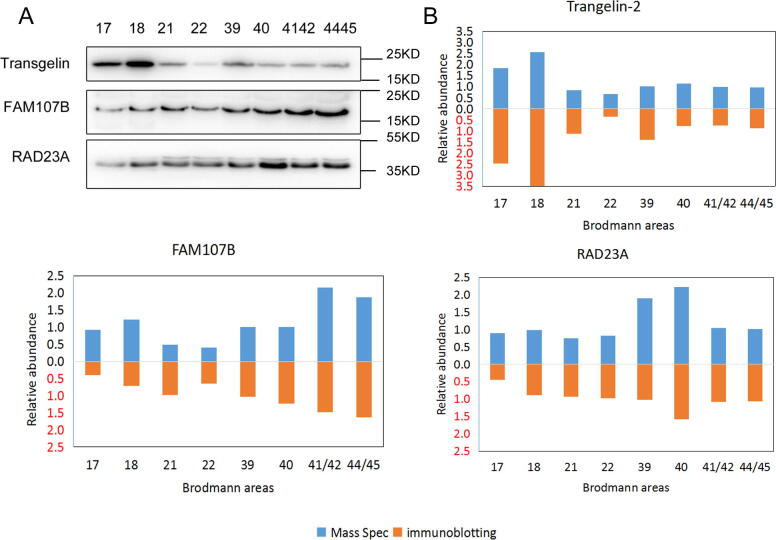
The Western Blot and IHC-Fr results of Brodmann area signature proteins. **A**. Western Blot results of three Brodmann area signature proteins (TAGLN, FAM107B, and RAD23A). **B**. Comparison between the immunoblotting results and iTRAQ results of the above proteins.

Additionally, we focused on BA-specific signature proteins (proteins with their abundances in one or two BAs more than two-fold higher than the median abundance) to investigate BA-specific functions. A total of 134 BA-specific signature proteins were identified based on the analog criteria suggested by Uhlen and colleagues [40] (Figure 4A; Table S4; see details in Materials and methods). These BA-specific signature proteins included several proteins closely associated with the corresponding brain functions. In the cingulate cortex, superoxide dismutase [Cu-Zn] (SOD1) was identified as a signature protein in the anterior cingulate cortex (BA32 and BA24). *SOD1* mutation interferes with GABAergic neurotransmission, leading to cortical neuronal loss in this brain area [41]*.* In the neocortex, FAM107B was identified as a signature protein in BA41/42 (auditory cortex) (Figure 4B). Knockout of *FAM107B* leads to hearing impairment in mice [42]. Three BA-specific signature proteins, TAGLN (BA17 and BA18), RAD23A (BA40), and FAM107B (BA41/42), were validated by Western blot (Figure 4B, Figure S9). Five BA-specific signature proteins, NEFH (BA4), PMP2 (BA4), CALB2 (BA28), TAGLN (BA17 and BA18), and FAM107B (BA41/42), were validated to be expressed at a high level in corresponding BAs in brain 2 (Table S5). Two primary visual cortex signature proteins, TAGLN and H1F0, were also found to be expressed at a high level in the primary visual cortex in Becky’s study [7] (Table S5).

Overall, these findings demonstrated intrinsic links between specialized functions of the brain clusters and BAs and molecular functions of the corresponding signature proteins, which may help to understand the mechanisms of physiological or pathophysiological functions of the brain.

### Molecular evidence of the connection between the motor cortex and anterior cingulate cortex

The anterior cingulate cortex is tightly connected to the motor cortex through neuronal cell projections, according to the results of fluorescence labeling imaging in rats [43]. In humans, Caruana et al. demonstrated that the anterior cingulate cortex plays an important role in the control of complex motor behaviors according to the results of intracortical electrical stimulation [44]. Molecular evidence of the connection between these two functional brain regions requires additional investigation.

**Figure 5:**
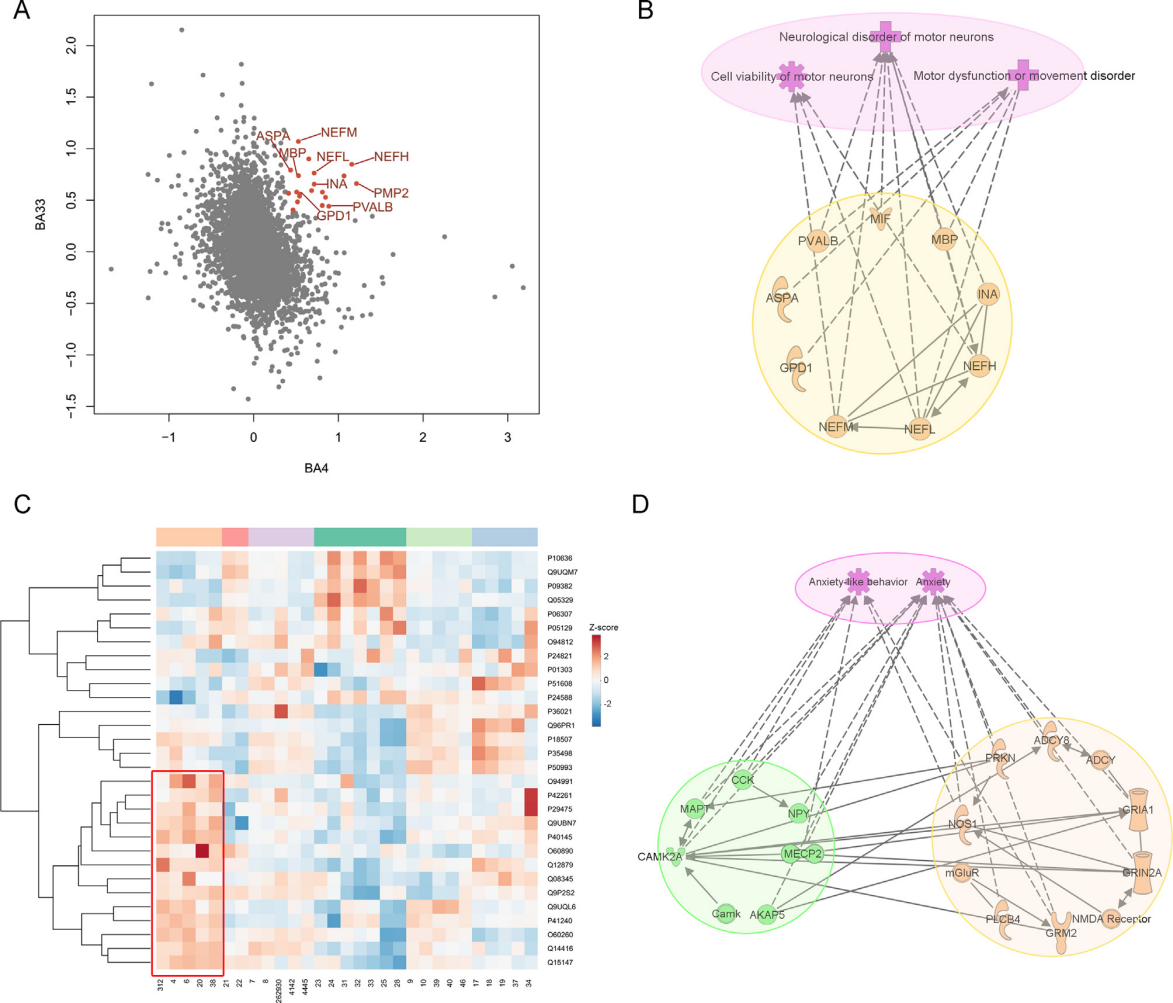
**Molecular annotation of the un-canonical functions of BAs** **A.** Scatter plot of protein expression levels (log_2_ FC) in BA33 compared with those in BA4. Proteins expressed at a high level in both BA4 and BA33 were highlighted in orange. **B.** The highly expressed proteins in both BA4 and BA33 were associated with motor neuron structure and functions. **C.** Clustering analysis of anxiety-related proteins. The proteins highly expressed in motor and sensory cluster were highlighted in red. **D.** Protein network of anxiety-related proteins. Orange, the proteins expressed at a high level in motor and sensory cluster. Green, the proteins expressed at a low level in motor and sensory cluster. FC, fold change.

Proteomic data obtained in the present study demonstrated that a series of proteins were expressed at a high level in the primary motor cortex (BA4) and anterior cingulate cortex (BA33) (Figure 5A; Table S6). Most of these proteins are associated with motor neuron structure and function (Figure 5B). Neurofilament proteins (NEFL, NEFM, NEFH, and INA) are the main components of motor neurons [45] and are necessary for the maintenance of the number, morphology, viability, and regenerative capability of motor neurons [46], [47], [48]. Myelin basic protein promotes the myelination of motor neurons [49]. MIF [50] and PVALB [51] are associated with amyotrophic lateral sclerosis, a neurodegenerative disease targeting motor neurons [52]. Thus, these proteins may represent the molecular basis of the functional connections between the primary motor cortex (BA4) and anterior cingulate cortex (BA33) and may also play a role in the motor functions of the anterior cingulate cortex.

### The motor and sensory cluster may be involved in anxiety

Anxiety disorder is a disease characterized by excessive and persistent worry and fear and is very common worldwide (lifetime prevalence is 5%–25% of the population), representing a substantial economic burden [53], [54]. The sensorimotor cortex has been reported to be associated with social anxiety disorder [55] and anxiety-related personality traits [56], according to the results of electrophysiological studies. However, the molecular basis of anxiety in the sensorimotor cortex requires further investigation.

Proteomic data obtained in the present study indicated that 30 of the 1241 proteins with interarea variability are involved in anxiety or anxiety-like behaviors according to Ingenuity Pathway Analysis (IPA) (Table S6). Clustering analysis indicated that nearly half (14, 46.6%) of these 30 proteins were expressed at a high level in the motor and sensory cluster (Figure 5C). Some of these 14 proteins, including ADCY8, GRIA1, GRIN2A, PLcB4, SLITRK5, NOS1, and GRM2, formed a protein interaction and regulation network and participated in the expression of anxiety and anxiety-like behaviors (Figure 5D). PlcB4 [57] and NOS1 [58] were expressed at a high level in the medial septum and the hippocampus (two anxiety behavior-related regions), respectively, and inhibition of these genes in the regions with high levels of expression increased anxiolytic effects in a mouse model. GRIN2A and GRIA1 are two important glutamate receptors involved in the function of the sensorimotor cortex [59] and also involved in anxiety. Knockout of *GRIN2A* [60] and *GRIA1* [61] causes a decrease in anxiety in mouse models. Thus, we hypothesized that these proteins are expressed at a high level in the motor and sensory cluster and may be involved in anxiety-related functions in the sensorimotor cortex, which may explain the molecular basis of anxiety-related processes in the sensorimotor cortex.

### Transcriptomic characterization of brain BAs

**Figure 6:**
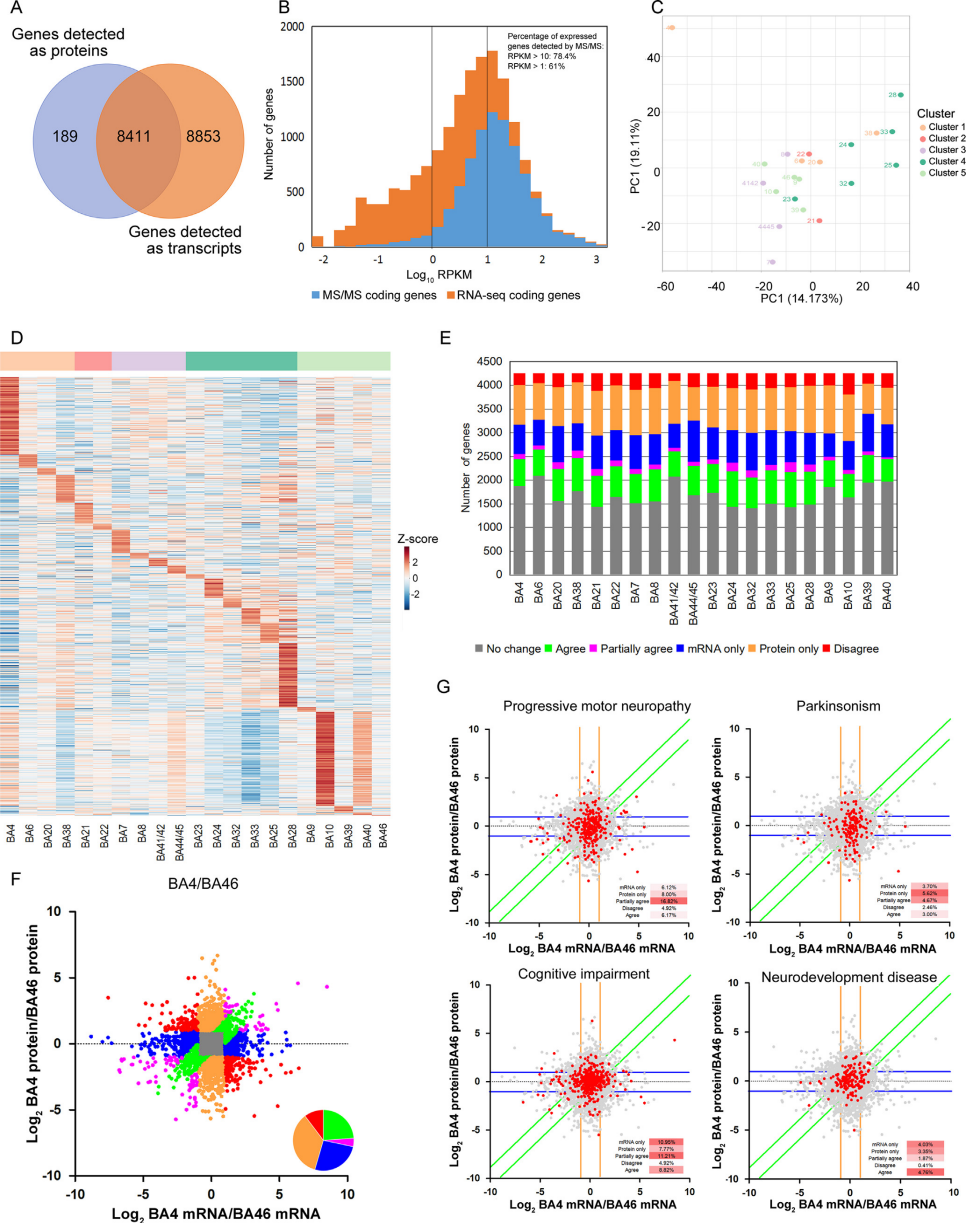
**Comparison of brain proteome and transcriptome** **A.** Venn diagram showing the overlap of proteins and mRNAs in BA46. **B.** Histogram showing the distribution of the gene expression at the mRNA and protein levels. Orange indicates genes identified at the protein level, and gray indicates genes only identified at the mRNA level. The MS/MS coding genes covered 61% of genes expressed with RPKM > 1 and 78.4% of genes expressed with RPKM > 10. **C.** Principal component analysis of mRNAs showed that the BA4 and cluster cingulate cortex were partially separated from the other BAs. **D.** Heatmap of the BA-specific signature genes in 27 BAs. The normalized protein expression value z-score represents the protein abundance. Red color indicates high abundance, and blue color indicates low abundance. **E.** The distribution of the relative dominance of “no change” (gray), “agree” (green), “partially agree” (purple), “protein only” (blue), “mRNA only” (orange), and “disagree” (red) genes. Genes were defined based on the consistency or inconsistency between the mRNA and protein measurements. “Agree” genes refer to the genes with the same direction of expression changes at both the mRNA and protein levels (FC > 2), and with the same magnitude of FCs (the variability at the protein level between BAs is less than 2-fold of that at the mRNA level). “Partially agree” genes refer to the genes with the same direction of expression changes (FC > 2), but with different magnitude of FCs at the mRNA and protein levels. “No change” genes refer to the genes with no expression change at both the protein and mRNA levels (FC ≤ 2) between BAs. “Protein only” genes varied between different BAs at the protein level (FC > 2), but not at the mRNA level (FC ≤ 2). “mRNA only” genes show expression changes at the mRNA level (FC > 2), but not at the protein level (FC ≤ 2). “Disagree” genes show opposite direction of expression changes between protein and mRNA. **F.** Scatter plot of log_2_-transformed mRNA and protein abundance FC between BA4 and BA46. Color codes are defined in (E). The inset pie charts illustrated the percentage of the green, purple, blue, orange, and red genes. **G.** Scatter plots of mRNA and protein FC for BA4/BA46 comparison, showing the position of gene products contained in ontological terms of interest. The inset small table showed the percentage of the genes of responding ontological terms in the “mRNA only”, “protein only”, “agree”, “partially agree”, and “disagree” categories. The colored lines indicate the threshold of “mRNA only” genes (orange), “protein only” genes (blue), and “agree” genes (green). RPKM, reads per kilobase per million mapped reads.

**Supplementary figure 10:**
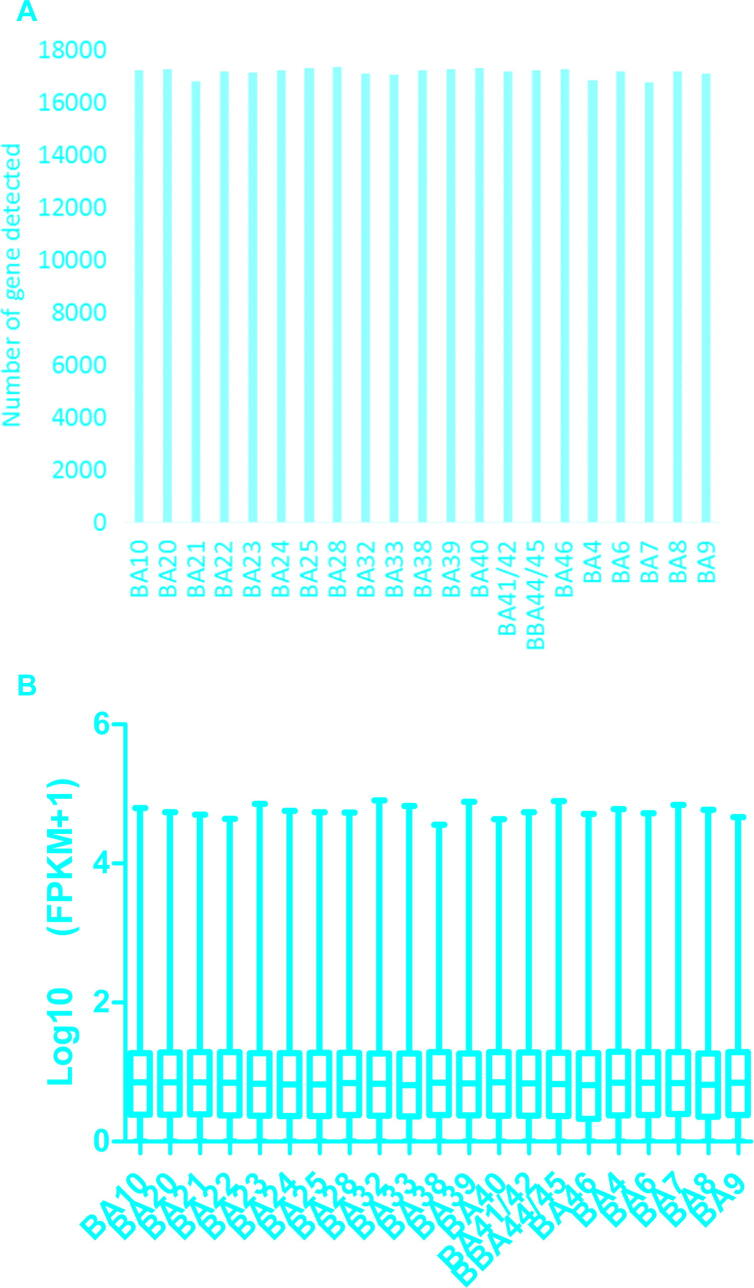
Quality control of RNA-seq. **A**. Number of gene detected in RNA-seq analysis of the 21 BAs. **B**. Box plot of gene expression level of 21 BAs. FPKM, Fragments Per Kilobase per Million.

The transcriptomes of brain BAs were explored to reveal the characteristics of the brain functions. The 29 BAs were analyzed by RNA-seq, and 21 samples passed the quality control [RNA integrity number (RIN) ≥ 6; 28S/18S ≥ 0.7; gene mapping ≥ 40%] and were used for further functional analysis. An average of 17,137 transcripts was detected based on the RNA-seq data (Table S7; Figure S10). Comparison of the brain proteome library and the transcriptomic database of BA46 identified 8411 transcriptomic entries (97.8%) for 8600 proteins detected by LC-MS/MS (Figure 6A). The proteomic data covered 48.8% of the transcriptomic data, covering 61% with expression level of reads per kilobase per million mapped reads (RPKM) > 1 and 78.4% with expression level of RPKM > 10 (Figure 6B).

A total of 17,091 genes were quantified in 21 BAs. Principal component analysis (PCA) of the transcriptomic data showed that BA4 (primary motor cortex) was different from other BAs, in agreement with the finding of the Allen Institute that a series of genes are specifically enriched in the primary motor cortex [1]. The cingulate cortex cluster tended to be partially separated from other BAs, which was partially consistent with the proteomic results. However, four functionally distinct clusters distinguished by proteomic analysis could not be found based on the transcriptomic data (Figure 6C).

The BA-specific signature genes at the transcriptomic level were used to explore BA-specific functions. A total of 1319 BA-specific signature genes were identified (Figure 6D; Table S7). Functional analysis indicated that BA-specific signature genes were closely involved in the functions of the corresponding BA. In the cingulate cortex, a total of 13 signature genes of BA28 were involved in memory function, in agreement with the functional characteristics of the ventral entorhinal cortex (BA28) [26] (Table S7). In the neocortex, motor neuron-related proteins (NEFH and NEFM) [46], [47] were identified as signature genes of the primary motor cortex (BA4), and were also expressed at a high level in BA4 in our proteomic data. In the auditory cortex (BA41/42), SMPX was identified as a signature gene. A previous study reported that mutation of *SMPX* was associated with X-linked deafness type 4 [62].

Expression levels of totally 4250 genes were quantified based on the data of transcriptomic and proteomic analyses in 21 BAs (Table S7). Gene products for all region pairs were classified based on the FC similarity between mRNA and protein (see details in Materials and methods). An average of 39.5% of the genes was classified into “no change” category (FC ≤ 2 for both protein and mRNA), and 17.4% of the genes were classified into the “agree” category [same direction of expression changes (FC > 2) and the same magnitude of FCs] or “partially agree” category [same direction of expression changes (FC > 2) and different magnitude of FCs]. In addition, 20.6% and 15.7% of the genes were considered “protein only” (FC > 2 at protein level only) and “mRNA only” (FC > 2 at mRNA level only), respectively. In contrast, 6.7% of the genes showed opposite direction of expression changes at the mRNA and protein levels (FC > 2; “disagree” category) (Figure 6E).

**Supplementary figure 11:**
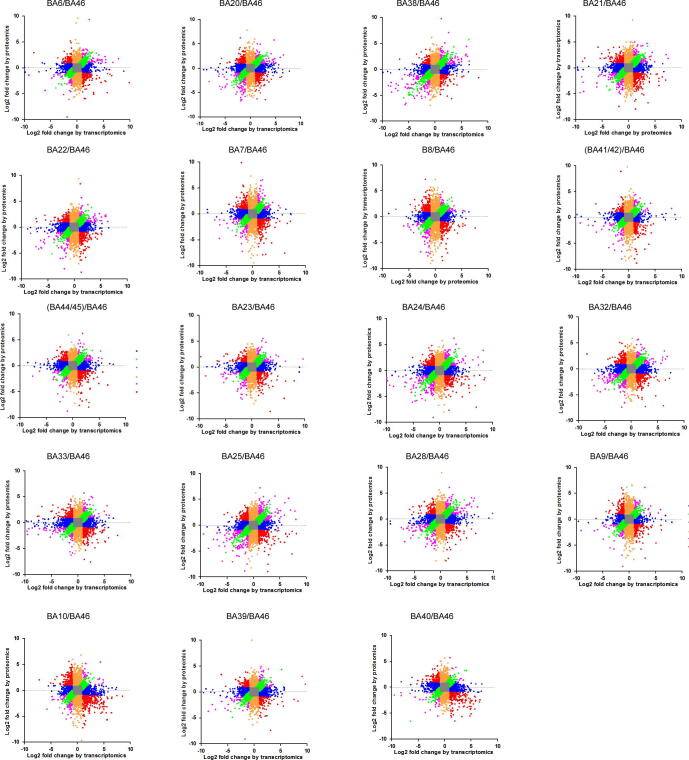
Scatter plots of mRNA and protein fold change of all regions compared with BA 46. These scatter plots are identically defined as those in Figure 6E.

To show the differences between transcriptome and proteome on brain functions, BA4 (primary motor cortex) and BA46 (DFC) were compared, and the scatter plots of the mRNA and protein FC between them are shown in Figure 6F (other BA pair comparison data are shown in Table S7 and Figure S11). According to IPA functional annotation of the differential gene products between BA4 and BA46, progressive motor neuropathy and Parkinsonism-related genes were enriched in “protein only” and “partially agree” categories, which could reflect the characteristics of the primary motor cortex. In contrast, the cognitive impairment and the depressive disorder-related genes showed a bias toward “mRNA only” category, reflecting the characteristics of the DFC. Additionally, the neurodevelopment-related genes were enriched in the “agree” category, consistent with Becky’s findings [7] (Figure 6G; Table S7).

## Discussion

In this study, we constructed a Chinese cerebral cortex proteome atlas of 29 BAs from a donor without neurological diseases. Similarities in the protein expression patterns indicated that these 29 BAs could be grouped into six clusters. The cluster- and BA-specific signature proteins can provide molecular evidence for annotation of cytoarchitecture, function, and disease vulnerability of these clusters or the corresponding BAs. All data are publicly available in the HBPA.

In this brain proteome atlas, BAs with similar protein expression patterns have similar functions, even though they are not spatially adjacent, which reflects functional parcellations. BAs within the cingulate cortex cluster and vision cluster are characterized by both spatial proximity and functional relevance. However, BAs within the auditory and Broca’s area cluster are not directly adjacent and are functionally connected (speech and auditory). These results indicated that the proteomic map mainly reflects the functional parcellations of the human cerebral cortex. Furthermore, the proteomic similarity of BAs may provide molecular annotations for functional connections between BAs. For example, BA38 (temporal pole) is an enigmatic region with largely unknown function [63]. The brain connectome indicated a functional connection between BA3/1/2-BA38 and BA21/22-BA38 [3]. The results of functional Magnetic Resonance Imaging (fMRI) experiments indicated that BA38 is involved in speech comprehension and considered a part of “extended Wernicke’s area” [64], [65]. A study on Braille reading in blind individuals using brain imaging methods indicated that BA38 could integrate sensory-motor and language comprehension [63]. In the present study, BA38 was characterized by protein expression patterns similar to both the sensorimotor cortex (BA3/1/2, BA4, and BA6) in the motor and sensory cluster and Wernicke’s area (BA21 and BA22). We suggest that BA38 may play integratory roles in both sensory-motor functions and language comprehension.

Additionally, the present study identified 474 cluster-specific and 134 BA-specific signature proteins. These proteins reveal intrinsic links between their molecular functions and specialized functions of the brain regions. The cingulate cortex contains an extremely large number of signature proteins compared to that of other neocortex regions. These signature proteins were enriched in metabolic pathways, mainly in glycolysis, glycogen turnover, and amino acid synthesis. Previous studies demonstrated that astrocytes had unique metabolic pathways of brain glucose metabolism compared to those in neurons [66]. First, glycolysis is predominant in astrocyte glucose metabolism. Second, the storage and turnover of glycogen are mainly concentrated in astrocytes. Third, tricarboxylic acid cycle-derived amino acids, such as aspartate, glutamate, glutamine, arginine, and GABA, are synthesized in astrocytes using astrocyte-specific enzymes [66]. The combination of previous reports and proteomic data obtained in the present study indicates the activation of astrocyte metabolism in the cingulate cortex, which may correspond to metabolic characteristics of the cingulate cortex. Additionally, the results of the present study suggest that some signature proteins in the cingulate cortex cluster participate in the pathogenesis of some neurological diseases, such as MAPT in Alzheimer’s disease and SYUA in Parkinson’s disease. Previous studies had shown that the accumulation of these two proteins in the cingulate cortex was a pathological feature of the corresponding diseases [28], [29]. Abnormalities in the cingulate cortex played a central role in the development of Alzheimer’s [24] and Parkinson’s diseases [67], [68]. These signature proteins may be investigated to determine molecular mechanisms of neurological diseases, especially in some specific brain regions. Thus, information about these proteins may substantially improve our understanding of the molecular basis of brain functions and pathogenesis.

Furthermore, the data of the present study provide molecular annotations of the non-canonical functions of brain regions. Molecular function analysis of these data provides a molecular basis for functional connections between the anterior cingulate cortex and motor cortex and may explain the molecular basis of anxiety in the sensorimotor cortex. These findings promote our understanding of the molecular mechanisms of the non-canonical functions of brain regions. Therefore, proteomic data obtained in the present study may be used to investigate new functions of BAs or new functional connections between BAs.

In the present study, the comparison of BA transcriptome and proteome indicated that the proteomic data could separate the cerebral cortex into 6 clusters, which were associated with BA’s function. However, transcriptomic data did not show a similar classification, which was consistent with Allen’s results [1]. The aforementioned results indicated that brain proteomic data might preferably reflect BA function. Functional analysis of the transcriptome suggested that it could also reflect the functional characteristics of BAs. 17.4% genes showed a similar differential expression trend as the protein (such as neurodevelopment), and some BA-specific signature genes (*NEFH* and *NEFL* in primary motor cortex) also showed consistent expression patterns as the protein. Moreover, 15.7% genes only differentially expressed in mRNA level, and some of which were associated with BA function (cognition impairment, depressive disorder in DFC). The aforementioned data demonstrated that transcriptome and proteome showed different characteristics of brain functions. According to previous studies [7], in neuron, mRNA and proteins might locate in different brain regions. Synapse proteins were transmitted from the nucleus into another BA through axon, so they could be detected in both BAs. However, mRNA was limited to the source BA containing cell body, so they were only identified in source BA. Thus, such genes would be differentially expressed only on mRNA levels. The aforementioned study could partially account for the differences between transcriptome and proteome.

This study established a Chinese proteome atlas of the brain; however, the following issues need to be resolved in future studies. First, we drafted a global multiregional proteomic map of the left hemisphere of a single brain. Previous proteomic and transcriptomic studies suggested that individual variations in age and gender were substantially lower than variations across brain regions [1], [7]. Proteome atlas generated in the present study can represent common characteristics of the normal human cerebral cortex to some extent; however, future studies should consider the inclusion of multiple individuals balanced with regard to gender, age, and brain hemisphere sampling and should use more sensitive and accurate proteomic technologies. Second, this pilot atlas includes only 29 BAs with important brain functions, and future studies should include additional important regions, such as the cerebellar cortex, hippocampus, and other important nerve nuclei, to generate a more comprehensive atlas.

## Materials and methods

### Tissue procurement

Samples from frozen postmortem human brain specimens were obtained following the SOP of the Human Brain Bank of China facilitated by the China Human Brain Bank Consortium (see Qiu et al. [9] for details on sample collection, handling, and preservation). Basic demographic information, medical information, and medical records of the donor were obtained. Brain 1 specimen was collected from a 54-year-old willed male donor who died of lung cancer and did not have any history of brain metastasis, trauma, or other diseases. Brain 2 was collected from a 93-year-old willed female donor who died of heart failure and did not have neurological diseases. The scalp was incised along the coronal plane from the ear to top of the skull and turned over to expose the skull. The skull was cut apart at the sagittal suture starting 1 cm above the eyebrows and continuing to the occipital protuberance, and the dura was removed. The cerebrum, brain stem, cerebellum, and part of the cervical cord were completely removed and temporarily preserved in cold saline. Twenty-nine samples of the cortex from various BAs were dissected from the left cerebral hemisphere and stored at −80 °C for protein extraction. Routine hematoxylin/eosin, silver, and immunohistochemical staining procedures for amyloid-beta, p-tau, and alpha-synuclein were performed in the visual cortex, inferior parietal lobule, superior and middle temporal gyri, medial frontal cortex, amygdala, anterior cingulate gyrus, hippocampus, basal ganglia, cerebellum, middle brain, cerebral pons, and medulla in the right cerebral hemisphere of the brain sample; the data were used for pathological diagnosis to exclude neurodegenerative diseases. According to the SOP [9], we performed HE staining of the brain areas, including BA40, BA24, BA17, and BA41/42/22, to confirm that brain is cancer-free.

### Sample preparation

A total of 29 brain tissue samples were selected for proteomic analysis (80 mg each). The samples were rinsed with PBS until washing fluid was clear, and each sample was lysed with lysis buffer (containing 8 M urea, 2.5 M thiourea, 4% CHAPS, 65 mM DTT, and 50 mM Tris-HCl) in a homogenizer on ice. The lysate was centrifuged at 14,000 r/min at 4 °C for 20 min, and the supernatant was collected. The Bradford method was used to measure the protein concentration. Proteins were digested by the FASP method, as previously described [69]. The peptides were extracted by an Oasis C18 extraction column and quantified by the BCA method.

### iTRAQ labeling and 1D off-line separation

Digested peptides were labeled with 8-plex iTRAQ reagent according to the manufacturer’s protocol (Sciex, Framingham, MA). The 28 BAs were randomly divided into 4 groups (Table S2) and labeled by iTRAQ 8-plex agent. In each group, BA46 was used as a common internal reference sample. The labeled samples within each experiment were mixed together. To reduce sample complexity, the peptide samples were fractionated through a high-pH Reverse Phase Liquid Chromatography (RPLC) system. The samples were reconstituted in 100 µl of buffer A (1 mM ammonia, pH 10) and loaded onto a column (4.6 mm × 250 mm, Xbridge C18, 3 μm, Waters, Milford, MA). The elution gradient was composed of 5%–25% buffer B (90% ACN, 1 mM ammonia, pH 10) at a flow rate of 0.8 ml/min for 60 min. 60 fractions were collected, dried, and resuspended in 0.1% formic acid; the fraction was pooled into 20 samples using a stepwise concatenation strategy to combine every 20th fraction (1, 21, 41; 2, 22, 42; …). A total of 100 fractions from one group of unlabeled samples and four groups of iTRAQ-labeled samples were further analyzed by LC-MS/MS.

### LC-MS/MS analysis

The pooled mixtures of iTRAQ**-**labeled samples were analyzed using a self-packed RP C18 capillary column (75 μm × 100 mm, 1.9 μm). The elution gradient was composed of 5%–30% buffer B (0.1% formic acid and 99.9% ACN) at a flow rate of 0.3 μl/min for 45 min. An LTQ Orbitrap Fusion Lumos instrument (ThermoFisher Scientific, Waltham, MA) was used to acquire raw data. The following parameters were used for MS data acquisition: top speed data-dependent mode (3 s) was used for a full scan; full scans were acquired in an Orbitrap at a resolution of 60,000; the MS/MS scans were acquired at a resolution of 15,000 with charge state screening (excluding precursors with unknown charge state or +1 charge state); 38% normalized collision energy in HCD mode; 30 s dynamic exclusion (exclusion size list 500); 1.6 Da isolation window. Each fraction from the iTRAQ-labeled sample was run twice. For label-free analysis, 32% normalized collision energy in HCD was used; other parameters were identical to the iTRAQ. Each fraction from the unlabeled sample was run three times.

### Database search

The MS/MS spectra were searched by Proteome Discoverer software (v2.1, ThermoFisher Scientific) using the SwissProt human database downloaded from the UniProt website (www.UniProt.org). Trypsin was selected as the cleavage specificity parameter, and allowed a maximum of two missed cleavage sites. For iTRAQ quantification, carbamidomethylation of cysteine and iTRAQ 8-plex labels were set as fixed modifications, and the oxidation of methionine, deamidation of asparagine and glutamine, carbamylation of lysine, and peptide N-terminus were used as dynamic modifications. The searches were performed using a peptide tolerance of 10 ppm and a production tolerance of 0.02 Da. A 1% false-positive rate at the protein level was set as a filter, and each protein had to contain at least 1 unique peptide. For label-free quantification, carbamidomethylation of cysteine was used as a fixed modification, and other search parameters were identical to the parameters used for iTRAQ quantification. The abundance of each protein was estimated by Progenesis software (Nonlinear Dynamics, v4.0), and the iBAQ value of each protein was calculated based on the abundance of a protein divided by the theoretical polypeptide number of the corresponding protein.

### Bioinformatics analysis of the iTRAQ data of 29 BAs

Each iTRAQ experiment contained seven BA samples and a common internal reference sample (BA46). In each experiment, the abundance ratios of the seven samples in BA46 were calculated, and the average protein abundance ratios of two technical replicates were used for further analysis. To ensure reliability of the data, the missing values in any two technical replicates of any group were excluded from subsequent analysis. The abundance ratios of proteins identified in all four experiments were merged into a single table in which each row represented a protein, and each column represented a BA. In the column for BA46, all protein ratios were filled with 1. Then, the ratios were log_2_-transformed and normalized to a column median of 0 and a row median of 0. Thus, the positive value was higher than the median, and the negative value was lower than the median. Through row-wise normalization, the expression pattern of each row/protein in all the samples could be better exhibited, and it would be helpful for following cluster analysis and signature proteins filtering.

For better cluster analysis and signature protein selection, the proteins without inter-area variability were filtered. The FCs of all proteins between all BA pairs were calculated. The distribution of log_2_ fold change showed that the and Q3 + 1.5×IQR (interquartile range) was 1.57. A strict filter (FC > 2) was used as the threshold of outliers, which would be helpful to signature proteins. To identify the differential proteins between Wernicke’s areacluster and auditory and Broca’s area cluster, a relatively relaxed threshold (FC = 1.5) was used to find more differential proteins, which would be helpful for pathway or function enrichment analysis.

Consensus clustering was performed using the consensus cluster plus package of R [11] to assess the similarities in the proteome profiles of BAs. *K*-means clustering with Euclidean distance was performed 1000 times on the subsampled data. The optimal number of clusters was selected using the consensus matrix, consensus CDF plot, and silhouette plot. The consensus matrix showed the best clustering results at *k* = 3. However, the CDF of consensus increased at greater *k* values. At *k* = 6, a relative increase in CDF reached a very small value. The silhouette plot at *k* = 6 showed a mean silhouette score of 0.72, and all scores were positive, indicating that clusters were well separated. Thus, BAs were clustered into six clusters.

Cluster signature proteins were identified by a biclustering algorithm known as ISA. ISA was implemented using the isa2 package for R [23]. Biclustering was restricted to up-regulated modules in both rows and columns with a score threshold of 0.7. For all six clusters, modules that included at least 80% BAs inside a cluster and no BAs outside of the cluster were selected. Proteins in the selected modules with scores that passed the threshold of 0.7 were considered signature proteins for this cluster.

An adequate cutoff ratio was determined to identify proteins overexpressed in specific BAs. The differences in the log_2_ protein abundance ratios between all BA pairs for all proteins were calculated, and the data were normally distributed with a mean of 0.00 and a standard deviation (SD) of 0.35. The log_2_ FC cutoff was then determined to be 1, which approximated the mean + 3 × SD. Thus, an area signature protein was defined as a protein with an abundance level in one or two particular BAs of at least 2-fold the median abundance level in all 29 BAs.

### Bioinformatics analysis

All proteins of interest were analyzed using the David database (http://david.ncifcrf.gov/) and compared with the whole human genome for GO enrichment analysis. For IPA analysis, the signature or differential proteins were evaluated using IPA software (Ingenuity Systems, Mountain View, CA), and the enrichment of the disease, function, and canonical pathway categories was analyzed according to the *P* value rankings. For reactome analysis, cluster signature proteins were mapped to the reactome database in the pathway category according to the *P* value rankings. For Kyoto Encyclopedia of Genes and Genomes (KEGG) analysis, cluster signature proteins were mapped to the KEGG database in the pathway category according to the *P* value rankings.

### IHC and Western blot

Western blot analysis of brain samples was performed to validate the proteomic quantitation of several selected candidate proteins (MAPT, SYUA, EPM2A, MRPS34, TAGLN, FAM107B, and RAD23A). Proteins extracted from the brain tissues were separated by SDS-PAGE and electro-transferred to a PVDF membrane (Millipore, Bedford, MA). The membrane was blocked with 2% (v/v) BSA for 2 h at room temperature and incubated with primary antibodies and a peroxidase-conjugated secondary antibody. The membranes were visualized using Immobilon Western chemiluminescent horseradish peroxidase substrate (Millipore), and the bands were analyzed by ImageJ software (version).

Formalin-fixed, paraffin-embedded brain tissue samples were used for IHC-Fr analysis. The tissue sections were deparaffinized and rehydrated in xylene and a graded ethanol series, and antigen retrieval was performed in a pressure cooker by boiling in sodium citrate buffer (pH 6.0) for 2 min. Then, endogenous peroxidases were blocked with 0.3% H_2_O_2_ for 15 min, and the samples were incubated in the presence of 2% fetal calf serum for 20 min. Primary antibodies were incubated at 4 °C overnight, and peroxidase-labeled polymer conjugated to anti-mouse, anti-rabbit, or anti-goat immunoglobulins was incubated for 1 h. The sections were counterstained with Mayer’s hematoxylin and dehydrated, and the images were acquired under a microscope.

Primary antibodies for Western blot and IHC-Fr against MAPT (rabbit monoclonal; Catalog No. ab76128), SYUA (rabbit monoclonal; Catalog No. ab138501), EPM2A (rabbit monoclonal; Catalog No. ab129110), MRPS34 (mouse monoclonal; Catalog No. HPA042112, Sigma), TAGLN (rabbit polyclonal; Catalog No. ab14106), and RAD23A (mouse monoclonal; Catalog No. ab55725) were purchased from Abcam (Cambridge, UK). Antibodies against FAM107B (rabbit polyclonal; Catalog No. ab122566) were purchased from Sigma-Aldrich (St Louis, MO).

### PRM analysis

Selected cluster and BA signature proteins were validated in 27 BAs of brain 2 by PRM. To estimate the quality of the data, we used the mixed sample as quality control (QC) during the analysis, before and after all samples, and every 7–8 samples. Two technical repeats of each sample were assayed. The average Pearson correlation coefficient of QC samples was 0.97, indicating the reproducibility of the QC.

The samples were analyzed on a C18 RP self-packed capillary LC column (75 μm × 100 mm). The elution gradient was composed of 5%–30% buffer B (0.1% formic acid, 99.9% ACN) with a flow rate of 0.5 μl/min for 45 min. An LTQ Orbitrap Fusion Lumos instrument was used to acquire raw data. Following parameters were used for MS data acquisition: PRM mode; full scans and MS/MS scans were acquired in Orbitrap at a resolution of 60,000 and 15,000, respectively; 32% normalized collision energy in HCD mode; 30 s dynamic exclusion; The isolation window was 4, and the schedule mode window was 7 min.

PRM data processing was performed by Skyline 19.1 software as previously described [69]. The peptide abundance was normalized to Total Ion Current (TIC) in each sample, which was extracted by Progenesis QIP software (Waters).

### RNA-seq and data processing

RNA-seq was performed by BGI cooperation (Shenzhen, China). For transcriptome data analysis, read counts were normalized using the median of ratios normalization method in DESeq2 to eliminate the variations in sequencing depth and RNA composition in the samples [70]. Normalized counts were logarithmically transformed with base 2. A total of 17,091 transcripts with read counts > 1 in at least 50% of the samples were retained for downstream analysis. Transcriptomic and proteomic data were matched by gene symbols.

### Comparison between the proteomic and transcriptomic data

Genes commonly quantified at the mRNA and protein levels were quantitatively compared. Both transcriptomic and proteomic datasets were log_2_-transformed by zero-mean normalization. Gene products for all possible region pairs were classified based on their FC similarity between mRNA and protein. Genes with the same direction of expression changes (FC > 2) at the mRNA and protein levels and with the same magnitude of FCs (the variability at the protein level between BAs was less than 2-fold of that at the mRNA level) were assigned to the “agree” category; genes with the same direction of expression changes (FC > 2) but with different magnitude of FCs (larger than 2-fold) at the mRNA and protein levels were assigned to the “partially agree” category. Genes that had no significant interregional changes at both the mRNA and protein levels (FC ≤ 2) between BAs were assigned to the “no change” category. Genes that had variable levels of corresponding proteins between various BAs (FC > 2) but did not vary at the mRNA level (FC ≤ 2) were assigned to the “protein only” category. Genes that had variable mRNA levels (FC > 2) but did not vary at the protein level (FC ≤ 2) were defined as “mRNA only”. Genes that showed opposite direction of expression changes at mRNA and protein levels were assigned to the “disagree” category.

## Ethical statement

Brain tissues were collected after obtaining consent from the donors and the next of kins using a signed written informed consent form, and the project was approved by the Institutional Review Board of the Institute of Basic Medical Sciences, Chinese Academy of Medical Sciences (Approval No. 009-2014).

## Data availability

The MS proteomic data have been deposited to the Proteome Xchange Consortium via the iProX partner repository (ProteomeXchange: PXD024613), and are publicly accessible at http://proteomecentral.proteomexchange.org. The raw transcriptomic database has been deposited in the Genome Sequence Archive [71] at the National Genomics Data Center, Beijing Institute of Genomics, Chinese Academy of Sciences / Chian National Center for Bioinformation (GSA: HRA000695), and are publicly accessible at http://ngdc.cncb.ac.cn/gsa.

## CRediT author statement

**Zhengguang Guo:** Formal analysis, Funding acquisition, Validation, Writing - original draft. **Chen Shao:** Methodology, Formal analysis, Software, Visualization, Funding acquisition, Writing - review & editing. **Yang Zhang:** Conceptualization, Writing - review & editing. **Wenying Qiu:** Resources, Investigation. **Wenting Li:** Resources. **Weimin Zhu:** Project administration. **Qian Yang:** Validation. **Yin Huang:** Software. **Lili Pan:** Visualization. **Yuepan Dong:** Visualization. **Haidan Sun:** Investigation. **Xiaoping Xiao:** Investigation. **Wei Sun:** Investigation, Project administration, Funding acquisition, Writing - review & editing. **Chao Ma:** Project administration, Funding acquisition. **Liwei Zhang:** Conceptualization, Project administration, Funding acquisition, Supervision. All authors have read and approved the final manuscript.

## Competing interests

The authors declare no competing interests.
